# From Traditional Application to Genetic Mechanism: Opinions on *Monascus* Research in the New Milestone

**DOI:** 10.3389/fmicb.2021.659907

**Published:** 2021-03-31

**Authors:** Jie Wang, Yueyan Huang, Yanchun Shao

**Affiliations:** College of Food Science and Technology, Huazhong Agricultural University, Wuhan, China

**Keywords:** red mold rice, *Monascus* species, secondary metabolism, development, ecological status

## Introduction

Red mold rice (RMR), the fermented product of *Monascus* strains, also called Hongqu, red fermented rice (RFR), and red yeast rice (RYR), has been used as a food coloring agent, food preservative, and traditional medicine for about 2,000 years in China (Chen et al., 2015). There are also historical records regarding the use of RMR in other oriental countries (Chen et al., 2015). Due to regional and cultural differences, there are many other names for RMR, such as beni-koji, red koji, or anka in Japan and Rotschimmelreis in Europe (Chen et al., 2015). Although RMR has a long history of consumption, we didn't know the *Monascus* strain until it was isolated from RMR in 1884 (Tieghem, 1884). Since then, scholars have gradually turned their attention to *Monascus* strain and its metabolic products (Patakova, 2013; Shao et al., 2014). *Monascus* species have great abilities to produce polyketides, such as well-known *Monascus* azaphilone pigments (MonAzPs), monacolin K (MK), and citrinin. Genomic information mining shows that *Monascus* spp. have great potential to produce multiple secondary metabolites (Chen et al., 2015), attracting more and more attention worldwide. Based on current progress of RMR and *Monascus* species, this opinion puts forward the issues that need to be further studied and discussed.

## The Categories of RMR and Their Applications

According to the applicable scope of RMR, it can be divided into three main categories, namely, color RMR (CRMR), functional RMR (FRMR), and brewing RMR (BRMR) (Feng and Yu, 2020). CRMR is the product rich in MonAzPs, which have been extensively used in the food industry as a natural food colorant. It is estimated that more than one billion people consume food containing MonAzPs-related products during their daily life (Yang et al., 2015). Currently, MonAzPs have become one of the fastest growing categories of natural food colorants in the Chinese market (Sun and Wang, 2019). Annual production of MonAzPs is estimated to exceed 20,000 metric tons in China alone (Yang et al., 2015). MonAzPs also possess a wide range of biological activities, making them potential as a functional food ingredient (Lin et al., 2017). In addition, MonAzPs have many promising applications in the cosmetics, textile, printing, and dyeing industries (Chen et al., 2019). FRMR is a product rich in MK. Since MK can reduce the synthesis of cholesterol by inhibiting the activity of HMG-CoA reductase to lower blood lipids, it has been developed as a blood lipid-lowering drug and health care product (Zhang et al., 2020). There are two forms of MK produced by *Monascus* strains containing the active β-hydroxy acid form that exerts pharmacological effects, as well as an inactive lactone form, which makes the side effects of MK less than currently reported statin-like drugs (Beltran et al., 2019; Zhang et al., 2020). BRMR is a kind of fermented product rich in esterification enzymes, which can be used to enhance the unique aroma components of food. In China, it is mainly used for the enhancement of the aroma of liquor and soy sauce (Xu et al., 2021).

## Current Research and Future Direction

Given large-scale utilization and economic importance of RMR, scholars make more efforts to study the *Monascus* species to improve the product quality. In an attempt to summarize the published articles, the themes regarding *Monascus* research can be clustered into six groups: (1) the classification and identification of *Monascus* strains; (2) the methods to improve useful metabolites but inhibit citrinin; (3) the isolation and identification of new metabolites; (4) the exploration of the functional activity of metabolites; (5) the biosynthetic pathway and genetic regulation of secondary metabolites and developmental process; and (6) genomic information mining. These studies have brought new life to the RMR. However, many issues about RMR and the *Monascus* species still need to be further discussed, which are summarized as followed.

### The Production of RMR

The method of producing RMR by solid-state fermentation (SSF) has continued from ancient times to today in China (Chen et al., 2015). This method looks simple and does not require large equipment, but it is time-consuming and relies on manual labor (Chen et al., 2015). So it is difficult to control RMR quality among different batches. Comparatively, liquid-state (submerged) fermentation (LSF/SF) has the characteristics of short period, not easy to be contaminated, and a higher degree of automation, so it is more and more favored by manufacturers. This has been practiced in MonAzPs production and also been considered to produce MK (Silveira et al., 2013; Feng et al., 2016). But the confusing problem is that the content of MK produced by LSF is much lower than the yield by SSF. This phenomenon has also been observed in antibiotic-producing bacteria *Cylindrocarpon* sp. LL-Cyan426 and *Acremonium* sp. LL-Cyan416, which was attributed to the interface of SSF providing various exceptional habitations, such as the gradient of pH, O_2_, and substrate concentrations as well as product concentrations for mycelia growth (Bigelis et al., 2006). If all kinds of RMR can be produced by LSF, the RMR fermentation industry will be bought to a new level.

Generally, *Monascus purpureus, Monascus ruber*, and *Monascus pilosus* are widely used as the producers of various types of RMR (Patel, 2016). In fact, since van Tieghem first isolated *Monascus* strains from RMR (Tieghem, 1884), more than 20 species of *Monascus* have been recorded (Li and Guo, 2003). These *Monascus* species come from a wide range of ecological environments, such as sand pine (Barnard and Cannon, 1987), the surface sediment samples of water (Cannon et al., 1995), soil (Celestino et al., 2014), honey, and nests of stingless bees (Barbosa et al., 2017), showing their high adaptability in complex environments. We know that *Monascus* strains for fermentation usually grow slowly and are easily contaminated. However, how do these *Monascus* strains survive in such a diverse natural environment? What is the ecological role of a *Monascus* strain in nature? There is no doubt that different environments should influence the genetic regulation and cell development even influence the secondary metabolites. So the exploration of these issues will help us to better understand and utilize *Monascus* resources.

### Correlation of Polyketide Biosynthetic Pathway and Their Regulation

Currently, MonAzPs, MK, and citrinin are the most well-known secondary metabolites produced by *Monascus* strains, and their biosynthetic pathways had been explained by several research groups (Fu et al., 2007; Chen et al., 2008, 2017; Balakrishnan et al., 2014; He and Cox, 2016). But there is a complicated relationship among these compounds. Usually, the production of MonAzPs was often accompanied by the contamination of mycotoxin (citrinin), meaning that MonAzPs high-producing strains usually have strong abilities to synthesize citrinin (Wang et al., 2012). For this reason, scholars made great efforts to decrease or eliminate the production of citrinin in RMR and the related products through optimization of fermentation parameters and strain screening. Yet, it is still not clear why citrinin and MonAzPs always coexist in RMR.

It was proposed that the initial synthesis of citrinin shared the same precursor and biosynthetic steps with MonAzPs (Hajjaj et al., 1999). With the application of molecular biology tools, it has been demonstrated that MonAzPs and citrinin have their own independent biosynthetic pathways, and genes encoding these two biosynthetic pathways form separate gene clusters located on two different chromosomes (Balakrishnan et al., 2014; Li et al., 2015; Ding et al., 2016; He and Cox, 2016; Chen et al., 2017), which presents a new idea for people to control citrinin in RMR through knocking out the genes responsible for citrinin synthesis. However, this didn't achieve the desired expectations but even led to some results contradictory to our current understanding of biosynthetic pathways with respect to MonAzPs and citrinin. An example is that the deletion of specific genes involved in citrinin synthesis decreased the production of citrinin and MonAzPs (Li et al., 2015); another example is that the deletion of genes in the MonAzPs biosynthetic pathways resulted in decreased MonAzPs and citrinin (Liang et al., 2017). Therefore, it is currently hard to explain the puzzling phenomenon of MonAzPs and citrinin from the perspective of biosynthetic pathways. Interestingly, strains with high MK production rarely produce citrinin but can produce visible MonAzPs. So it is necessary to explore the relationship among these three polyketides at the genetic level.

The biosynthesis of secondary metabolites are regulated by multiple levels. It is well-known that certain regulatory factors, such as global regulator MrLaeA (Liu et al., 2016), components of G-protein signaling pathway, including MrFlbA (a regulator of G-protein alpha subunit) α, β, and γ subunits (Yang et al., 2012; Lei et al., 2019), and response regulator MrSkn7 have been demonstrated to modulate the production of MonAzPs, MK, and citrinin (Shao et al., 2016). But it is a remarkable fact that the regulation trend of these regulators on MonAzPs and citrinin is consistent. Are there any regulators that provide reverse regulation of the production of MonAzPs and citrinin? This is also an interesting question worthy of investigation.

### The Relationship Between Development and Secondary Metabolism

Secondary metabolism is always coupled with developmental processes (Chen et al., 2019). At present, the regulators reported in *Monascus* strains have played important regulatory roles in the production of MonAzPs and citrinin, and usually affected their growth, sexual, and asexual development (Yang et al., 2012; Liu et al., 2016; Shao et al., 2016; Lei et al., 2019). Do these regulators affect the secondary metabolic process by influencing the developmental process, or vice versa? This is a common problem with other filamentous fungi and a great challenge. If this question can be explained clearly, it will play an important guiding role in the rational improvement of industrial strains.

## Summary

Although RMR has been used as a traditional fermented food for nearly 2,000 years, we still lack a deep understanding of *Monascus* species at the genetic level. With the application of genome information mining and modern biotechnology, the genetic information of *Monascus* species will be continuously deciphered, which will help us make better use of *Monascus* resources.

## Author Contributions

JW and YH collected data and drafted part of the manuscript. YS drafted the manuscript and revised it. All authors commented on the manuscript.

## Conflict of Interest

The authors declare that the research was conducted in the absence of any commercial or financial relationships that could be construed as a potential conflict of interest.
